# Treatment of osteonecrosis of the femoral head using autologous cultured osteoblasts: a case report

**DOI:** 10.1186/1752-1947-2-58

**Published:** 2008-02-25

**Authors:** Seok-Jung Kim, Won-Jong Bahk, Cheong-Ho Chang, Jae-Deog Jang, Kyung-Hwan Suhl

**Affiliations:** 1 Department of Orthopedic Surgery, College of Medicine, The Catholic University of Korea, Seoul, Korea; 2 Central Research Institute, SW-Cellontech, Seoul, Korea

## Abstract

**Introduction:**

Osteonecrosis of the femoral head is a progressive disease that leads to femoral head collapse and osteoarthritis. Our goal in treating osteonecrosis is to preserve, not to replace, the femoral head.

**Case presentation:**

We present the case of a patient with bilateral osteonecrosis of the femoral head treated with autologous cultured osteoblast injection.

**Conclusion:**

Although our experience is limited to one patient, autologous cultured osteoblast transplantation appears to be effective for treating the osteonecrosis of femoral head.

## Introduction

Osteonecrosis of the femoral head is a progressive disease that leads to femoral head collapse and osteoarthritis [1]. A number of surgical procedures have been developed to preserve the femoral head, however, there is no single treatment method which completely cures this debilitating disease.

Bone regeneration by autogenous cell transplantation is one of the most promising treatment concepts currently being developed, as it eliminates the problems of donor site morbidity for autologous grafts, the immunological problems of allogenic grafts, and loosening of implants in total joint arthroplasty.

## Case presentation

**Figure 1:**
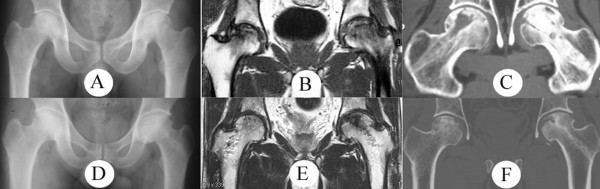
**A) Preoperative AP radiograph of both hips shows round cystic change with a sclerotic rim and no femoral head flattening in either femoral head.** B) Superior delineations of the necrotic areas in both femoral heads are seen on a T1-weighted, coronal, preoperative MRI image. C) Post-operative CT image of both femoral heads shows core decompression sites in both femoral heads and allograft impaction of the left femoral head. D) Both hip AP radiographs, E) MRI and F) CT images, were taken five years following surgery.

A 31-year old man was admitted with symptoms of acute joint pain of three weeks' duration in both hips. The patient had no specific past history of disease and his laboratory findings were normal. Plain radiographs (Fig. 1A) and MR examination (Fig. 1B) revealed Ficat II osteonecrosis of both femoral heads. The left femoral head was treated by allograft immediately after core decompression, while the right side was treated by injection of autologous cultured osteoblasts for four weeks after the core decompression (Fig. 1C).

Follow-up CT obtained one year following treatment, demonstrated that the right femoral head had bone reformation in multiple necrotic areas, that the femoral head was still in optimal condition, and that the left head showed absorption of the grafted bone as well as disease progression.

Radiographs obtained five years following surgery showed evidence of remodeling as well as maintenance of the right femoral head, but the left femoral head showed slight irregularity, sclerotic changes, and osteophyte formation (Fig. 1D). On both the MRI (Fig. IE) and the CT (Fig. 1F) images obtained five years following surgery, the right femoral head showed nearly complete healing of the necrotic lesions, while the necrotic lesions and subchondral bone breakage were still demonstrated in the left femoral head. At the time of five-year follow-up, the patient did not complain of right hip joint pain and had considerable restoration of a full range of joint motion, however, he still complained of intermittent pain and slight limitation of motion in the left hip.

### The isolation of bone marrow stromal cells and the culture of osteoblasts

Approximately 3 ml of bone marrow aspirated from the patient's posterior iliac crest, were added to a container filled with 30 ml of 10% FBS -α MEM (Sigma Chemical Company, St. Louis, MO, USA) and 350 units of heparin; the mixture was then taken to a laboratory. The mixture was centrifuged at 4°C, 472 g for 10 minutes after which the supernatant was discarded and 20 ml of culture medium was added to the remaining pellets. The mixture was filtered (Falcon, Franklin Lakes, NJ, USA), 10 ml of the medium were added per T-75 culture flask (Corning Science Products, Corning, NY, USA) and culture was initiated[2]. The incubator (Automatic CO2 Incubator, Forma Scientific Inc, Marietta, OH, USA) was maintained at 37°C with 5% CO2. The next day, 50 μg L-ascorbic acid (Sigma)/10 ml and dexamethasone 10^-7^M were added to facilitate cell differentiation into osteoblasts. The cell culture condition was evaluated by a light microscope, and the culture medium was changed on the fifth day of culture, after which the culture medium was changed every three days with the subsequent addition of L-ascorbic acid. On the fourteenth day of culture, NBT-BCIP (nitro blue tetrazolium chloride – 5-bromo-4-chloro-3-indolyl phosphate) staining was performed to confirm activation of the alkaline phosphatase. Twenty-four days after beginning the culture, Alizarin red staining was performed to detect newly produced calcium, and it was thus confirmed that most of the cultured cells were osteoblasts. Approximately four weeks after beginning the culture, the medium was removed and the cells were washed with 5 ml 0.02% trypsin-ETDA (Gibco BRL, Gettysburg, PA, USA). 3 ml of 0.02% trypsin-ETDA was again added and the cells were incubated for five minutes. The trypsin-ETDA activity was stopped by adding 3 ml of culture medium, and all contents were collected in a conical tube and were centrifuged at 4°C, 265 g, for 6 minutes. The supernatant was removed, and the precipitate was collected. After adjusting the cell count to 1.2 × 10^7^/ml, the cells were used in the transplant.

### Surgical technique

Under local anesthesia, the patient was placed on a fracture table in a lateral position with the affected hip upside. A 19-G spinal needle was attached to a 2-ml-syringe which contained the cultured osteoblasts which were then inserted into the deepest portion of the core decompression site with the guidance of a C-arm fluoroscopic image intensifier. Two ml of cell mixture were slowly injected with progressive withdrawal of the spinal needle into the junction of the femoral head and neck. After completing the injection, a slight compression force was applied to the injection site for hemostasis and the lateral position was maintained for 10 minutes. After core decompression surgery, the patient did not put weight on both hips for six weeks, after which he gradually advanced during the next eight weeks to full weight-bearing.

## Discussion

Experimentally, bone marrow stromal cells have been known to have the potential to differentiate into osteoblast, chondroblast, fibroblast or adipocyte, depending on the environment of the adjacent tissues [3]. However, as the number of bone marrow stromal cells in bone marrow is extremely low, cell culture is considered to be a prerequisite for its clinical utilization [4].

To our knowledge, until recently there have been no clinical attempts to treat osteonecrosis, or long-term follow-up of the treatment of osteonecrosis, using cultured autologous osteoblasts. If cultured autologous cells are successfully used for this treatment, some problems related to bone graft techniques might be overcome, such as donor site morbidity in autografts [5,6] and immunological problems in allografts [7].

A drawback to this technique can be the two-stage surgery. However, the second surgery consists only of injection under local anesthesia. During this procedure, our patient was very comfortable and without pain.

Autologous cultured osteoblast injection is based on bone marrow injection which is supported by the theory that osteoprogenitor cells in bone marrow induce and facilitate bone formation [8]. Bone marrow injection is performed independently or in combination with a bone graft procedure. This procedure is simple and has no donor site morbidity or complications. However, as the amount of aspiration volume at one site is limited and the number of bone forming cells is small [9], it is assumed that the culturing of cells and their subsequent transplantation is the most feasible method to overcome such a problem, and by the transplantation between different species using mediators, successful results have been reported [10].

We consider that the osteoblast transplantation we administered to our patient was successful as it relieved the patient's symptoms and provided considerable restoration of a full range of joint motion. In contrast to the traditional bone graft technique in which considerable time is required for the resorption of transplanted bone and for the reformation process [11], osteoblast transplantation appears to be helpful in readily incorporating the immature bone tissue formed by the injected osteoblasts into the adjacent tissue without need for the resorption or the reformation process. In addition, the organizing hematoma developed at the decompression site seems to act as a scaffold for the injected autologous cultured osteoblasts, thus appearing to be better for bone regeneration than any of the other artificial carriers.

## Conclusion

Although to date our experience is limited to one patient, autologous cultured osteoblast transplantation appears to be effective for treating osteonecrosis of the femoral head.

## Competing interests

The author(s) declare that they have no competing interests.

## Authors' contributions

SK was involved in collecting patient details, reviewing the literature, and drafting the manuscript as the main author. WB and JJ were involved in reviewing the literature and proofreading the manuscript. K-HS performed the final revisions of the manuscript. CC is the senior author and was responsible for final proofreading of the article.

All authors read and approved the final manuscript.

## Consent

The authors confirm that written informed consent was obtained from the patient for publication of the manuscript. A copy of the written consent is available for review by the Editor-in-Chief of this journal.
